# Coronary atherosclerosis: plaque volume determines long-term prognosis

**DOI:** 10.1093/ehjci/jeag040

**Published:** 2026-02-03

**Authors:** Axel Schmermund, Thomas Voigtländer, Philipp Breitbart

**Affiliations:** Cardioangiologisches Centrum Bethanien, CCB, Im Prüfling 23, 60389 Frankfurt am Main, Germany; Cardioangiologisches Centrum Bethanien, CCB, Im Prüfling 23, 60389 Frankfurt am Main, Germany; Cardioangiologisches Centrum Bethanien, CCB, Im Prüfling 23, 60389 Frankfurt am Main, Germany

**This editorial refers to ‘The time-varying prognostic value of stenosis and plaque burden in coronary artery disease’, by R.A. Jukema *et al*., https://doi.org/10.1093/ehjci/jeag022.**

Historically, invasive cardiologists have focused on the detection of high-grade stenoses and the need for revascularization. The extent of coronary atherosclerosis was determined as ‘1- to 3-vessel disease’, depending on the number of stenotic major coronary arteries. Although there was certainly awareness of the diffuse nature of coronary atherosclerotic disease and the limitations of visualizing only the contrast-enhanced lumen, it was the classic pathology studies by Glagov and colleagues and the growing availability of intravascular ultrasound, IVUS, in the 1990’s that led to an enhanced understanding of the dynamic atherosclerotic changes in the vessel wall and their prognostic significance.^1,2^ The advent of coronary CT-angiography (CTA) has allowed for a much broader application of these concepts, swinging back and forth between the focus on severe stenosis or rather underlying plaque volume.

The current paper by Jukema *et al.*^3^ describes the outcome of patients examined in Amsterdam, Netherlands, and Turku, Finland, between 2007 and 2016. All patients underwent CTA using a CT-scanner with ≥64 slices for suspected coronary artery disease (CAD). Artificial intelligence (AI)-based software was used to analyse the CT data, generating (i) maximum diameter stenosis (%) and (ii) percentage atheroma volume (PAV), representing overall plaque volume (mm^3^) normalized to total vessel volume. A total of 2819 patients without known CAD were included with a mean age of 62 years, 45% male. All-cause mortality and non-fatal myocardial infarction (MI) were the endpoints documented over a median of 6.9 years, the longest follow-up being 8 years. Overall, 319 patients (11%) underwent revascularization as a consequence of CTA (‘early revascularization’). Death or MI related to early revascularization were not considered endpoint events. Between 6 months and 8 years, 235 endpoints were recorded, i.e. 160 deaths and 75 MIs. Patients with endpoints had greater maximum stenosis degree (median, 45% vs. 21%) and greater plaque volumes (median PAV, 12% vs. 3%). Whereas diameter stenosis was associated with a clearly elevated hazard ratio within the first year of follow-up (6–12 months), the association with events declined steadily thereafter. During the first 3–4 years, it approximately halved with regard to MIs and even became almost neutral regarding overall mortality after 2 years. The hazard ratio associated with PAV significantly increased between years 1 and 2 and thereafter remained relatively stable for both subgroups of endpoint events. This was statistically independent of risk factors or early revascularization status, lending itself to the following interpretation: The increase in risk conferred by the CT parameters varied between 2% and 4% per year and changed over time as described above; diameter stenosis losing and PAV gaining in importance. Thus, stenosis severity and PAV appeared to capture different temporal phases of coronary risk, stenosis reflecting short-term risk and plaque volume rather cumulative atherosclerotic risk over years (*Figure 1*). Overall, this view supports a dynamic concept of risk trajectories derived from CTA.

**Figure 1:**
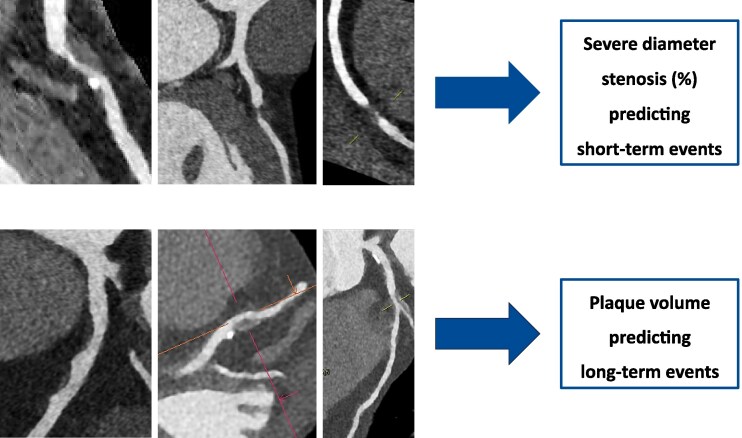
Time-varying prognostic significance of stenosis versus plaque volume.

How can these findings be explained? Apart from stenosis and plaque volume, it might be helpful to bring in a third variable, i.e. plaque vulnerability. While there may not be the one vulnerable plaque, and processes outside local plaque formation substantially contribute to the occurrence of clinical events, it is still worthwhile deliberating plaque characteristics which have been identified by CTA as prognostic determinants. Two features stand out, that is, (i) non-calcified plaque volume and in particular the subclassification of low attenuation plaque (corresponding to large atheroma volume) as well as (ii) high-grade stenosis.^4,5^ Their prognostic meaning exists independently of each other and of other characteristics associated with plaque vulnerability such as napkin ring sign, spotty calcification, or expansive remodelling. The prognostic importance of plaque volume and stenosis has been confirmed by invasive imaging studies.^6,7^

Certainly, viewed prospectively, plaques associated with little or low-grade stenosis can cause events because of rupture or erosion. However, on the path to rupture or erosion, they grow and become stenotic.^8,9^ The mechanisms of plaque progression and stenosis formation are exactly those eventually leading to clinical events, namely inflammation and repeated subclinical thrombosis.^9^ Accordingly, stenosis grade and clinical events are two sides of the same medal. Whereas stenosis grade may still be low 1 year before the event, it almost invariably appears to have progressed to high-grade in the days preceding the acute event. Stenosis grade and clinical events are both associated with accelerated pathophysiologic activity and plaque growth.

In view of the relationship between plaque progression and vulnerability, the detection of high-grade stenoses inherently identifies patients who have undergone a destabilization process that significantly increases their risk of an acute clinical event if not reversed by optimal medical treatment or perhaps revascularization. Adopting this concept, stenoses can be understood as the vanguard of plaque progression and vulnerability and possibly the last stage before clinical misfortune takes its course. Under this assumption, it makes sense that in the current paper, stenoses are the major predictor of events within the first year. After having undergone treatment, most patients will achieve ‘pacification’ of the atherothrombotic and inflammatory mechanisms, so that the prognostic implications of baseline stenoses subside. Those with a large PAV, however, still are at greater risk than others. They may need intensified therapy which may not always be successful. Consequently, in the long run, PAV becomes more prognostically important than stenoses. PAV may represent the persistence of disease activity even after the early stenosis-associated risk has been attenuated.

How robust are the findings by Jukema *et al.*? They used state of the art CT-technology and artificial intelligence for quantitative analysis of coronary anatomy, plaque burden, and degree of stenosis. Follow-up time and number of events were sizeable, and only five patients were lost to follow-up.^3^ However, they performed an observational analysis using clinical data, and the potential for bias must be taken into account. Obviously, one can expect that the diagnosis of a high-grade stenosis did lead to clinical workup and usually interventional or surgical revascularization. It appears somewhat self-evident that early on, stenoses were prognostically more relevant. Yet, events during the first 6 months were not included, and sensitivity analyses either adjusting for early revascularization status or excluding all patients with early revascularization did not indicate a change of the results.^3^ Another point of critique could be the nature of the events during follow-up. There were 75 MIs and 160 deaths. Of the latter, 50 or 60 may have been related to coronary atherosclerosis, so that the predictive ability of the CT parameters may have been measured by using a diluted reference including competing non-cardiovascular risks, thus attenuating discriminatory performance. On the other hand, these are real-world data. The events must have meant a heavy burden for the patients and their relatives and friends, and it would certainly have been desirable to avoid or postpone them. In this sense, the paper presents a valuable analysis of the varying prognostic potential CTA may offer. This holds true even though the current database did not allow for evaluating the potential impact the CT findings may have had on initiating or withholding medical treatment.

Another important methodological aspect relates to image acquisition and the generalizability of AI-based plaque quantification. It is well recognized that quantitative plaque assessment by AI-based software may vary depending on scanner hardware, image quality, and reconstruction techniques. Consequently, direct comparability of PAV and stenosis metrics across different scanners may be limited. This raises the question whether the relative relationship between stenosis severity and plaque burden was fully homogeneous between study sites and scanner generations. Moreover, it remains open if quantitative thresholds and prognostic relationships can be applied to contemporary high-end CT-scanner techniques or alternative AI-based software solutions.

Jukema *et al.* are to be congratulated for presenting a meticulous and thoughtful analysis. Their observational database may have some limitations, and it is obvious that further evidence is needed before we can be certain about the time-varying prognostic interplay between coronary stenoses and plaque volume. Nevertheless, the central message is clear, and it corroborates previous reports on the prognostic significance of the two parameters^4,10,11^: CTA provides for easily available prognostic parameters with notable predictive value, i.e. stenosis and plaque volume. We believe the way does indeed not lead to ever more complicated measurements of high-risk plaque characteristics such as the napkin ring sign. Rather, we should focus on the reliable quantification of the two most critical parameters, stenosis, and plaque volume.

## Data Availability

Data sharing is not applicable to this article as no new data were created or analysed in this study.
